# A Malignant Duo: Mixed Medullary and Follicular Variant Papillary Thyroid Cancer

**DOI:** 10.7759/cureus.67231

**Published:** 2024-08-19

**Authors:** Anwar Alshaakh Mohd Mari, Joane Titus, Vania Zayat, Mustafa Kinaan

**Affiliations:** 1 Internal Medicine, Hospital Corporation of America (HCA) University of Central Florida (UCF), Florida, USA; 2 Internal Medicine, University of Central Florida (UCF) College of Medicine, Orlando, USA; 3 Pathology, Orlando Veterans Affairs Medical Center, Orlando, USA; 4 Pathology, University of Central Florida (UCF) College of Medicine, Orlando, USA; 5 Endocrinology, Hospital Corporation of America (HCA) Florida Osceola Hospital, Kissimmee, USA

**Keywords:** history of hashimoto's disease, a malignant duo, follicular thyroid cancer, medullary, mixed medullary-follicular carcinoma

## Abstract

Medullary thyroid cancer (MTC) is a relatively rare thyroid malignancy, constituting a small percentage of all thyroid cancer cases. Even more rare is the occurrence of mixed MTC and papillary thyroid cancer (PTC), found in a very small fraction of MTC cases. These cancers originate from different cell types with distinct developmental origins. The coexistence of MTC and PTC in the same patient raises questions about whether this occurrence is merely coincidental or if there is an underlying genetic link. We present the case of a woman with metastatic mixed MTC and PTC. A 61-year-old woman with a history of Hashimoto's disease was found to have bilateral thyroid nodules; the largest (1.7 cm) was in the right lobe. This nodule met fine needle aspiration (FNA) biopsy criteria and was found to have a follicular neoplasm of undetermined significance. The patient elected to pursue total thyroidectomy instead of lobectomy given the presence of bilateral nodules. Postoperative pathology showed mixed medullary carcinoma (pT3b) and follicular variant papillary thyroid microcarcinoma (pT1a) involving the right lobe with positive anterior and posterior margins and lymphovascular invasion. Preoperative calcitonin was not checked. However, post-thyroidectomy calcitonin was 1599 pg/mL. She underwent central and right lateral neck dissection which showed 27 out of 35 lymph nodes were positive for malignancy. Postoperative calcitonin dropped to 38.7 pg/mL. She then established care in our endocrine clinic. Screening for pheochromocytoma and primary hyperparathyroidism was normal. She underwent external beam radiation of the neck. A year after her initial surgery, her neck ultrasound and computed tomography (CT) studies show no signs of local or distant anatomic recurrence. Her thyroglobulin level remains undetectable, carcinoembryonic antigen (CEA) within normal range, and calcitonin stable at about 20 pg/mL. She is on levothyroxine 100 mcg daily with thyroid-stimulating hormone (TSH) at a suppression goal of <0.1 mIU/L. Mixed PTC and MTC is poorly studied due to its rarity. The origin of these mixed tumors is unclear, but some suggest that they arise from neoplastic changes of remnant multipotent cells in the thyroid. While patients with PTC often have a favorable prognosis following surgical therapy, MTC has a more aggressive course. We suggest monitoring patients like ours for both MTC and PTC, as if present in isolation. Our case highlights the clinical aspects of this condition and our current knowledge of its pathophysiology.

## Introduction

Medullary thyroid cancer (MTC) is an uncommon and challenging malignancy [1]. It is rare and only constitutes 5-10% of all thyroid malignancies [2]. Papillary thyroid cancer (PTC) differs significantly from MTC in terms of cellular origin, histological appearance, clinical course, biological behavior, and prevalence. The histogenesis origin and potential molecular mechanisms behind the development of mixed MTC-PTC remain unknown [3]. PTC and MTC can coexist in a mixed tumor that exhibits characteristics of both cancer types, known as mixed medullary-follicular thyroid cancer (MMFTC). MMFTC is rare and only found in less than 1% of MTC cases. These malignancies arise from different cell origins, follicular cells of the endoderm, and parafollicular C cells of the neural crest [4]. It is not clear if the coexistence of these tumors in the same patient is a coincidence or if there is a common genetic culprit.

A study published in 2024 found that somatic RET mutations are common in MMFTC, guiding targeted treatments like selpercatinib, which has shown promising results in clinical trials. The prognosis and treatment strategies are influenced by the predominance of the medullary carcinoma component and the presence of lymph node and distant metastases [5].

We present the case of a lady with metastatic MMFTC who had a successful response to surgery and radioiodine therapy.

This case was presented at the American Association of Clinical Endocrinology (AACE) Annual Meeting 2023, and the abstract was published in Endocrine Practice [6].

## Case presentation

Patient history

A 61-year-old woman with a history of Hashimoto's disease for more than five years was found to have a thyroid nodule on examination by her primary care physician.

Initial workup and surgery

**Figure 1:**
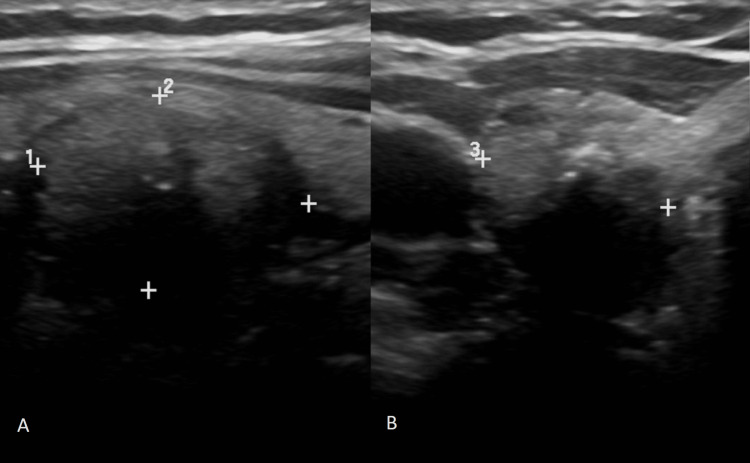
Ultrasound images of the thyroid gland showing a hypoechoic, heterogeneous, solid nodule measuring 1.72×1.22×1.20 cm in the right lobe with microcalcifications ((A) sagittal view, (B) transverse view). These features are indicative of potential malignancy, warranting further investigation through FNA biopsy. FNA: fine needle aspiration

The patient had a neck ultrasound which showed bilateral thyroid nodules, the largest (1.7 cm) in the right lobe (Figure 1). This nodule met fine needle aspiration (FNA) biopsy criteria and was found to have a follicular neoplasm of undetermined significance. Molecular testing was not performed on the FNA specimen. Based on these results, the primary care physician referred the patient to a surgeon for further evaluation. The patient elected to pursue total thyroidectomy instead of lobectomy given the presence of bilateral nodules. Of note, the patient was not evaluated by an endocrinologist prior to proceeding with surgery. 

Postoperative pathology evaluation

**Figure 2:**
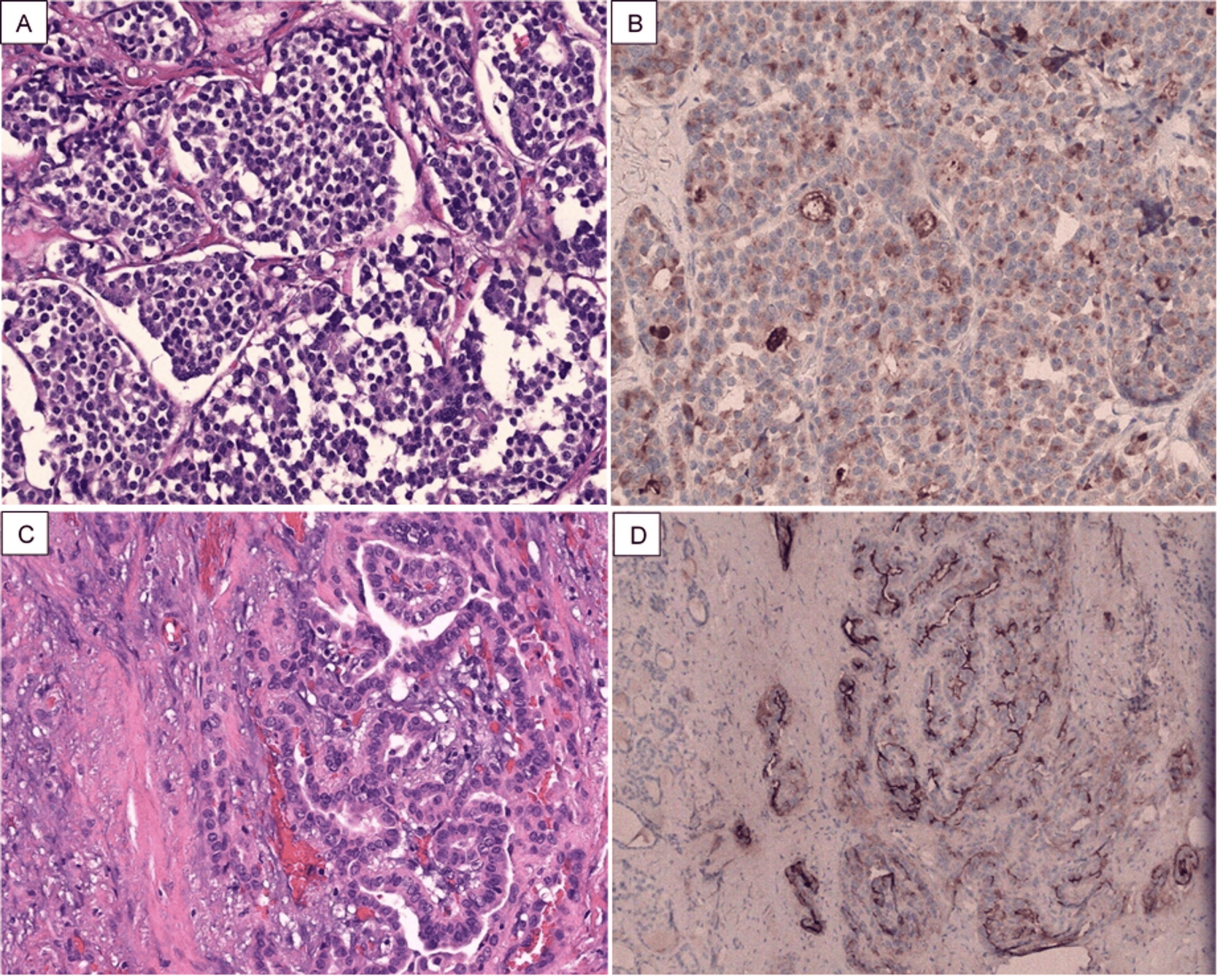
Histopathology of the thyroid gland. (A) Multiple solid nests of plasmacytoid cells and polygonal cells with coarse chromatin and eosinophilic cytoplasm consistent with medullary carcinoma. (B) Calcitonin IHC positive, confirming the presence of medullary carcinoma. (C) Papillae with fibrovascular core lined by cuboidal cells with nuclear clearing, overlapping, grooving, and pseudo-inclusions consistent with papillary carcinoma. (D) HBME-1 (IHC) positive, supporting the diagnosis of papillary carcinoma. IHC: immunohistochemistry

Postoperative pathology showed mixed medullary carcinoma (pT3b) and follicular variant papillary thyroid microcarcinoma (pT1a) involving the right lobe nodule with positive anterior and posterior margins and lymphovascular invasion. Postoperative histopathologic examinations showed features of MTC with positive calcitonin staining (Figure 2A, 2B) and features of follicular variant PTC with positive HBME-1 staining (Figure 2C, 2D). The tumor also had positive TTF-1, chromogranin, and carcinoembryonic antigen (CEA) staining. Based on these findings, the patient was diagnosed with MMFTC. 

Endocrinology evaluation and further management

Following the diagnosis of malignancy in postoperative pathology, the patient was referred to our endocrine team for further management. Although preoperative calcitonin was not checked by the surgical team, we checked post-thyroidectomy calcitonin, and it was 1599 pg/mL (normal range: <5 pg/mL), indicating residual MTC and neck metastases. She underwent central and right lateral neck dissection, which showed metastases in one lymph node in level 6 and 27 out of 35 lymph nodes in levels 2-4. Postoperative calcitonin dropped to 38.7 pg/mL. She underwent external beam radiation of the neck. Screening for pheochromocytoma and primary hyperparathyroidism was normal which ruled out multiple endocrine neoplasia (MEN) syndrome.

Follow-up

A year after her initial surgery, her neck ultrasound and computed tomography (CT) studies showed no signs of local or distant anatomic recurrence. Her thyroglobulin level remains undetectable, CEA within normal range, and calcitonin stable at about 20 pg/mL. She is on levothyroxine 100 mcg daily with thyroid-stimulating hormone (TSH) at a cancer suppression goal of <0.1 mIU/L.

## Discussion

MMFTC is a rare and under-researched entity. Medullary features can make this cancer aggressive and more difficult to diagnose and treat versus the most common variant, PTC. The pathology of these malignancies lies within their cellular origin. Follicular cells give rise to follicular thyroid cancers and PTC which represent approximately 85% of thyroid cancer cases, while MTC, which is extremely rare, arises from neuroendocrine parafollicular C cells comprising close to 4% of all thyroid cancers [7].

RAS mutations are commonly observed in follicular-patterned carcinoma. In contrast, genetic and molecular studies have shown the involvement of the RET proto-oncogene in hereditary MTC and, less frequently, in its sporadic form. The findings indicate that patients with RAS mutations have a better prognosis compared to those with RET mutations or those with no detectable mutations [8].

The presence of both MTC and PTC in the same gland is a rare occurrence and accounts for nearly 2.7-12.3% of all MTC cases, with an incidence of 0.1 per 100,000 individuals per year [9,10]. These tumors can coexist as two separate lesions with different histologies, cellular features, and origins (MTC-PTC). Rarer is the entity of a single tumor exhibiting features of mixed medullary thyroid cancer and papillary thyroid cancer (MMPTC) or MMFTC, as is the case with our patient [8]. While some data examining MTC-PTC features have been reported, very scarce information about MMFTC and MMPTC forms is available. This is most likely due to the inadequate distinction between these different forms. A Korean study of 53 cases of mixed tumors found that 19% of the cases had normal tissue separating the medullary and papillary cancers which is consistent with MTC-PTC rather than a tumor displaying both features [11].

The median age of patients with MTC-PTC is about 54 years, which is older than those with MTC (median age 44.5) but younger than those with PTC (median age 57.3) [9]. MTC-PTC affects twice as many females as males. Risk factors include being female, as in our case above, family history, exposure to ionizing radiation, alcohol and tobacco use, and obesity.

A study utilizing the National Cancer Database (NCDB) of 296,101 patients reported that 0.14% of cases were MMPTC and 0.04% were MMFTC. Compared with PTC, MMFTC and MMPTC patients were older, had more comorbidities, and had less nodal but more distant metastases. Although the 10-year overall survival rate for MMPTC was lower than PTC, it was higher than MTC. Interestingly, MMFTC had the worst survival rate, even worse than MTC [12].

Clinical features of MTC-PTC often include thyroid nodule or neck mass, which can be accompanied by flushing, diarrhea, and elevated calcitonin levels. Ultrasonography is the primary method for detection, identifying nodules with or without capsular invasion and microcalcification.

Diagnosing mixed thyroid tumors can be challenging. FNA results can be misleading or inconclusive, such as the case of a 43-year-old woman reported by Tohidi et al. where FNA showed anaplastic features, but postoperative pathology was consistent with MMFTC [13]. In another case, a 57-year-old had FNA results suggestive of MTC. However, postoperative pathology of the tumor showed intermingled components, one with negative thyroglobulin but positive calcitonin immunoreactivity consistent with MTC, while the other had the opposite pattern which is consistent with PTC [4].

The best clinical approach to mixed PTC and MTC tumors has not been established. We suggest treating patients like ours for the most aggressive form, MTC with surgical management, and monitoring for both MTC and PTC, as if present in isolation.

Due to the rarity and complexity of this cancer type, it is critical that patients diagnosed with MMFTC receive care from a medical team with experience in treating thyroid cancers, particularly in cases containing mixed histology.

PTC is mostly treated with total thyroidectomy followed by radioiodine ablation, while MTC is not radioiodine avid and requires a much more extensive surgical approach due to the high risk of recurrence. Patients with MTC must undergo total thyroidectomy with central neck dissection. Lateral neck dissection would also be performed if cervical metastasis is confirmed or highly suspected in MTC cases. External beam radiation might be necessary in MTC patients with cervical metastases or residual or recurrent disease. If MTC recurrence occurs, then chemotherapeutic agents (such as vandetanib and cabozantinib), further surgical or radiation therapy, or enrollment in medical trials for novel therapies can be considered. Given the aggressive behavior of MMFTC based on the literature, we recommend that it is treated in the same manner as MTC through extensive surgical resection and neck exploration. Our patient had significant cervical metastatic disease and underwent external beam radiation accordingly. Patients with MMFTC had significantly worse overall survival and worse prognosis in comparison to those with differentiated thyroid carcinoma [12].

Outcome and follow-up

A year after her initial surgery, the patient continues to be followed up with our endocrinology clinic and has no signs of anatomic or biochemical recurrence. Her neck ultrasound and CT studies show no signs of local or distant anatomic recurrence. Her thyroglobulin level remains undetectable, CEA within normal range, and calcitonin stable at about 20 pg/mL. She is on levothyroxine 100 mcg daily with TSH at a cancer suppression goal of <0.1 mIU/L.

## Conclusions

As noted earlier, MTC is a rare type of thyroid malignancy, accounting for a small fraction of all thyroid cancers. Mixed cases involving both MTC and PTC are even more rare, observed in a very limited number of instances. These cancers develop from different types of cells, with MTC originating from parafollicular C cells and PTC from follicular cells. The simultaneous presence of both types of tumors in a single patient raises questions about whether this is a coincidental occurrence or if there is an underlying genetic cause.

Our case sheds light on the MMFTC and rare MMPTC. Large population studies and clinical trials of patients with mixed PTC and MTC are needed to better understand its epidemiology, response to different therapies, and prognosis. This would in turn help devise an evidence-based approach to treatment. 

This case also emphasizes the importance of involving an endocrinologist when evaluating thyroid nodules, especially in patients with inconclusive FNA biopsy results. A more thorough preoperative evaluation with serum calcitonin level and molecular testing of FNA biopsy could've helped diagnose MTC prior to proceeding with surgery, hence saving this patient the need for repeat neck surgery.

Additionally, it is crucial to treat MMFTC tumors as aggressive variants and monitor patients closely with neck ultrasounds and tumor markers (calcitonin, thyroglobulin, CEA) to detect early signs of recurrence and improve survival.
